# Supplementary material for: Licuri Kernel (Syagrus coronata (Martius) Beccari): A Promising Matrix for the Development of Fermented Plant-Based Kefir Beverages
Source: Foods. 2024 Jun 27;13(13):2056. doi: 10.3390/foods13132056 (PMC11240999; doi:10.3390/foods13132056)
Supplement: Supplementary file 1 [file foods-13-02056-s001.zip › foods-3040197-supplementary.pdf]

Licuri kernel (*Syagrus coronata (martius) beccari*): a promising matrix for the development of fermented plant-based kefir beverages

Janaína de Carvalho Alves [1,*], Carolina Oliveira de Souza [1,2,3,*], Livia de Matos Santos [2], Suelen Neris Almeida Viana [2], Denilson de Jesus Assis [4,5], Pedro Paulo Lordelo Guimarães Tavares [2], Elis dos Reis Requião [3], Jéssica Maria Rio Branco dos Santos Ferro [6] and Mariana Nougalli Roselino [3,6]

[1] Northeast Biotechnology Network, Institute of Health Sciences, Federal University of Bahia, Av. Reitor Miguel Calmon, s/n, Salvador 40231-300, Brazil
[2] Graduate Program in Food Science, Faculty of Pharmacy, Federal University of Bahia, R. Barão de Jeremoabo, 147, Salvador 40170-115, Brazil; liviamatos@ufba.br (L.d.M.S.); suelen.neris@ufba.br (S.N.A.V.); pp.lordelo@gmail.com (P.P.L.G.T.)
[3] College of Pharmacy, Federal University of Bahia, R. Barão de Jeremoabo, 147, Salvador 40170-115, Brazil; elis.requiao@ufba.br (E.d.R.R.); mariana.roselino@ufba.br (M.N.R.)
[4] School of Exact and Technological Sciences, Salvador University, Av. Tancredo Neves, 2131, Salvador 41820-021, Brazil; denilson.assis@unifacs.br
[5] Graduate Program in Chemical Engineering (PPEQ), Polytechnic School, Federal University of Bahia, R. Prof. Aristídes Novis, 2, Salvador 40210-630, Brazil
[6] Postgraduate Program in Microbiology (PPG-MICRO), Institute of Biology, Federal University of Bahia, R. Barão de Jeremoabo, 668, Salvador 40170-115, Brazil; jessica.ferro@ufba.br
* Correspondence: janaina.carvalho@ufba.br (J.d.C.A.); carolods@ufba.br (C.O.d.S.)

Supplementary material

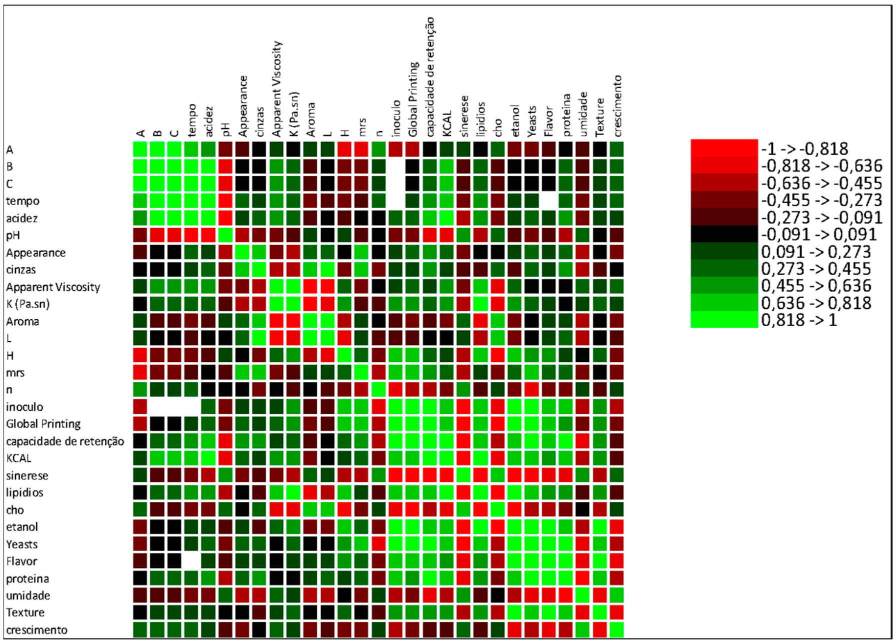

Figure S1. Image of the Spearman test correlation matrix (p<0.05); Alpha significance level=0.05

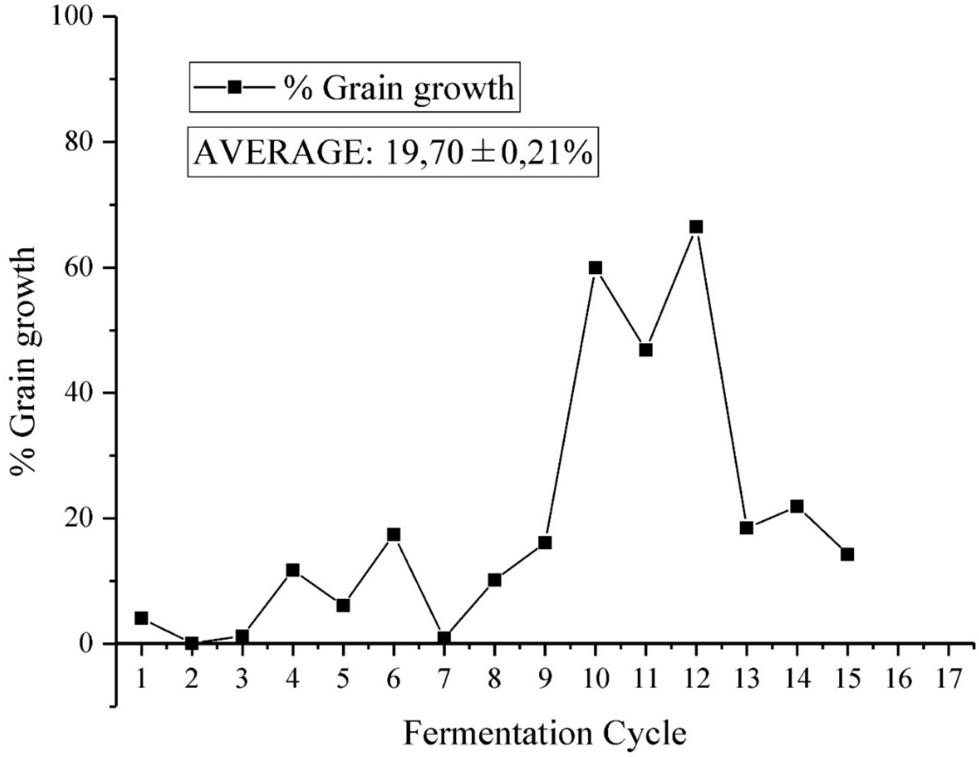

Figure S2. Growth rate of water kefir grains during 15 cycles of activation and cultivation in 10% sucrose solution

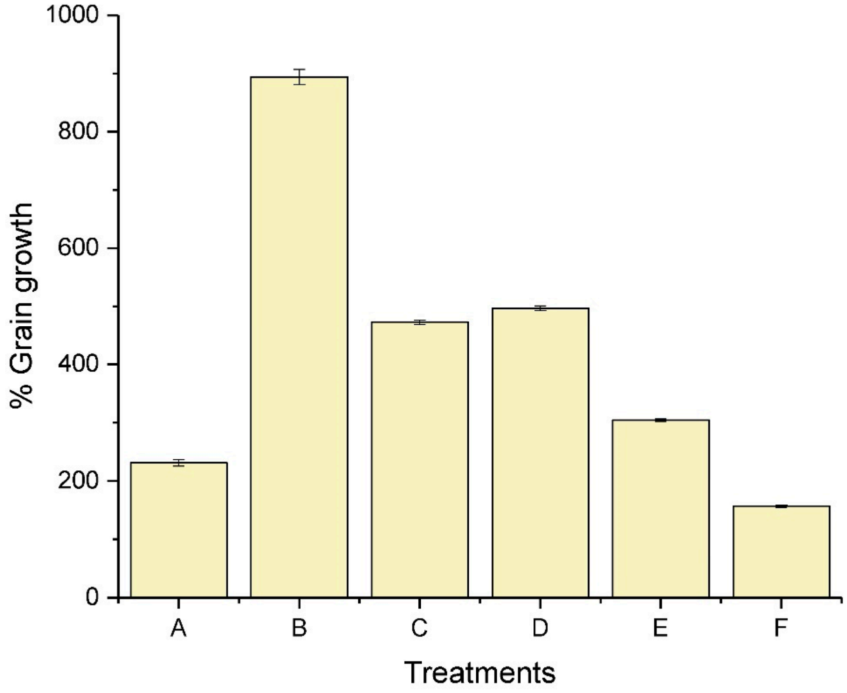

Figure S3. Percentage of growth of kefir grains adapted into a plant-based licuri drink
(A) Fermented for 24 hours with 1% inoculum; (B) Fermented for 48 hours with 1% inoculum; (C) Fermented for 24 hours with 2.5% inoculum; (D) Fermented for 48 hours with 2.5% inoculum; (E) Fermented for 24 hours with 5% inoculum; (F) Fermented for 48 hours with 5% inoculum

Table S1. Cosine of Squares (ACP)

Parameters	Axes				
	F1	F2	F3	F4	F5
Grain growth	0,009	0,362	**0,509**	0,026	0,095
Acidity	**0,784**	0,178	0,010	0,023	0,005
pH	**0,730**	0,077	0,061	0,063	0,069
LAB	0,181	0,206	0,134	**0,244**	0,235
Ethanol	**0,567**	0,189	0,033	0,209	0,001
Syneresis	**0,893**	0,043	0,016	0,041	0,006
WRC	**0,876**	0,075	0,028	0,002	0,019
Pa	**0,381**	0,042	0,188	0,283	0,106
Apparent Viscosity	0,331	0,005	**0,606**	0,005	0,052
Carbohydrate	**0,693**	0,199	0,055	0,019	0,035
Protein	**0,566**	0,094	0,139	0,201	0,000
Yeasts	**0,614**	0,009	0,156	0,078	0,142
JAR (Acidity)	**0,486**	0,217	0,019	0,240	0,039
JAR (sweet)	**0,584**	0,200	0,002	0,213	0,001
l*	0,006	0,079	**0,772**	0,142	0,002
a*	0,000	**0,933**	0,059	0,000	0,008
b*	0,176	**0,806**	0,006	0,008	0,004
c*	0,165	**0,816**	0,008	0,007	0,004
h*	0,086	**0,793**	0,105	0,005	0,011

Values in bold correspond for each variable to the factor for which the squared cosine is the largest

Table S2. Microbiological criteria evaluated for the fermented beverages developed

**Formulations**	**Salmonella spp. / 25g**	**Total coliforms (30ºC) NMP/ml**	**Thermotolerant coliforms (45ºC) NMP/ML**	***E. coli (CFU/ml)***	**Molds and yeasts (CFU/ml)**
**A**	Absent	$3,18\pm0,16^b$	$<3,0^a$	Absent	$1,40\pm0,22^a$
**B**	Absent	$4,16\pm 0,99^a$	$<3,0^a$	Absent	$1,20\pm0,27^a$
**C**	Absent	$3,06\pm0,13^b$	$<3,0^a$	Absent	$1,40\pm0,89^a$
**D**	Absent	$3,00\pm0,00^b$	$<3,0^a$	Absent	$1,10\pm0,55^a$
**E**	Absent	$3,24\pm0,25^b$	$<3,0^a$	Absent	$1,20\pm0,27^a$
**F**	Absent	$3,00\pm0,00^b$	$<3,0^a$	Absent	$1,20\pm0,45^a$

(±) standard deviation of the triplicate analyses used to calculate the means; Different lowercase letters indicate significant differences (Tukey $p < 0.05$) depending on the treatment used for the time evaluated; (A) Fermented for 24 hours with 1% inoculum; (B) Fermented for 48 hours with 1% inoculum; (C) Fermented for 24 hours with 2.5% inoculum; (D) Fermented for 48 hours with 2.5% inoculum; (E) Fermented for 24 hours with 5% inoculum; (F) Fermented for 48 hours with 5% inoculum. Molds and yeasts: mold count, without considering typical yeast colonies, since it is a fermented food with a natural presence of yeast
